# Proteome analysis of vaccinia virus IHD-W-infected HEK 293 cells with 2-dimensional gel electrophoresis and MALDI-PSD-TOF MS of on solid phase support N-terminally sulfonated peptides

**DOI:** 10.1186/1743-422X-8-380

**Published:** 2011-08-01

**Authors:** Sebastian Bartel, Joerg Doellinger, Kai Darsow, Daniel Bourquain, Rainer Buchholz, Andreas Nitsche, Harald A Lange

**Affiliations:** 1 Friedrich-Alexander University Erlangen-Nuremberg, Institute of Bioprocess Engineering, Henkestraße 91, 91052 Erlangen, Germany; 2 Robert Koch-Institute, Center for Biological Security 1, Nordufer 20, 13353 Berlin, Germany

**Keywords:** viral Proteomics, Vaccinia Virus, MALDI-PSD-TOF MS, SPITC

## Abstract

**Background:**

Despite the successful eradication of smallpox by the WHO-led vaccination programme, pox virus infections remain a considerable health threat. The possible use of smallpox as a bioterrorism agent as well as the continuous occurrence of zoonotic pox virus infections document the relevance to deepen the understanding for virus host interactions. Since the permissiveness of pox infections is independent of hosts surface receptors, but correlates with the ability of the virus to infiltrate the antiviral host response, it directly depends on the hosts proteome set. In this report the proteome of HEK293 cells infected with Vaccinia Virus strain IHD-W was analyzed by 2-dimensional gel electrophoresis and MALDI-PSD-TOF MS in a bottom-up approach.

**Results:**

The cellular and viral proteomes of VACV IHD-W infected HEK293 cells, UV-inactivated VACV IHD-W-treated as well as non-infected cells were compared. Derivatization of peptides with 4-sulfophenyl isothiocyanate (SPITC) carried out on ZipTipμ-C18 columns enabled protein identification via the peptides' primary sequence, providing improved s/n ratios as well as signal intensities of the PSD spectra. The expression of more than 24 human proteins was modulated by the viral infection. Effects of UV-inactivated and infectious viruses on the hosts' proteome concerning energy metabolism and proteins associated with gene expression and protein-biosynthesis were quite similar. These effects might therefore be attributed to virus entry and virion proteins. However, the modulation of proteins involved in apoptosis was clearly correlated to infectious viruses.

**Conclusions:**

The proteome analysis of infected cells provides insight into apoptosis modulation, regulation of cellular gene expression and the regulation of energy metabolism. The confidence of protein identifications was clearly improved by the peptides' derivatization with SPITC on a solid phase support. Some of the identified proteins have not been described in the context of poxvirus infections before and need to be further characterised to identify their meaning for apoptosis modulation and pathogenesis.

## Background

Despite remarkable progress in the control and treatment of infectious diseases, the problem of emerging and re-emerging pathogens is likely to be one of the main issues of medical science and public health in the twenty-first century [1]. In this respect viral diseases are of particular concern, because advances in the field of antiviral drugs have lagged behind those regarding bactericidal drugs and antibiotics. It was shown by the emergence of the severe acute respiratory syndrome (SARS) that new members of neglected virus families can cross into humans from unsuspected reservoirs, making rapid advances in our understanding of virus-host dynamics necessary [2]. In this regard poxviruses are of particular importance. Despite the success of the WHO-led smallpox eradication programme, other related human-pathogenic poxviruses remain a considerable threat. In particular, the 2003 outbreak of human monkeypox virus in the United States of America pointed out the imminence posed by zoonotic pox infections originating from animal-borne viruses [3]. Moreover, an increasing number of human cowpox virus infections, especially affecting younger people lacking smallpox vaccination, have been reported in Europe in recent years [4]. In addition to the threat of zoonoses, the relevance of a deeper understanding of the interactions of poxviruses with their host cells is pointed out by considering the classification of smallpox as a category A bioterrorism agent by the Centers for Disease Control and Prevention (CDC), especially since no acceptable treatment is available and the immunity in the population is declining [5].

In contrast to many other viruses, the ability of poxviruses to productively infect a given cell is not determined at the level of specific host receptors, but is regulated downstream of virus adsorption to the cell surface and virus entry by intracellular processes which for the most part are unexplored [6-10]. Since the permissiveness of orthopoxviruses is therefore dependent on the hosts proteome set, the identification of cellular proteins affected by the protein products of the so-called viral host-range genes with proteomic approaches will be a starting point for further functional protein analysis. The characterization of the Vaccinia Virus (VACV) virions' proteome has enlightened the protein equipment of the virus at the entry stage of infection [11]. Furthermore, virus-host dynamics have been analysed by yeast two-hybrid (Y2H) studies to identify interaction partners [12]. No closer look at the dynamic changes of the human proteome during a VACV infection has been taken yet on a global scale.

In this report an infection of human embryonic kidney (HEK) 293 cells with well-studied VACV was chosen as a model system. Uninfected HEK 293 cells serve as a control, while VACV-infected HEK 293 cells inactivated with UV enable the study of the influence of the virions' proteins on the cellular proteome. The investigation of infection effects on the expression of the cellular proteome was carried out in the late phase of virus replication. An offline-coupling of two-dimensional sodium dodecylsulfate polyacrylamide gel electrophoresis (SDS-PAGE) and Matrix Assisted Laser Desorption/Ionisation Time of Flight Mass Spectrometry (MALDI-ToF MS) was used in a bottom-up approach. In-gel trypsinated proteins from relevant spots of the 2-D gels are unambiguously identified via Mascot database search of the peptide mass fingerprint (PMF) recorded in positive ion reflectron mode of the MALDI-ToF MS. Identification rate was improved by obtaining additional sequence data of selected peptides in post source decay (PSD) mode, whereas a considerably advanced data quality was achieved by N-terminally derivatising the peptides on a ZipTipμ-C18 solid phase support with 4-Sulfophenyl isothiocyanate (SPITC) prior to MS measurement.

## Results

### PSD spectra of SPITC derivatized peptides improve protein identification efficiency

**Figure 1:**
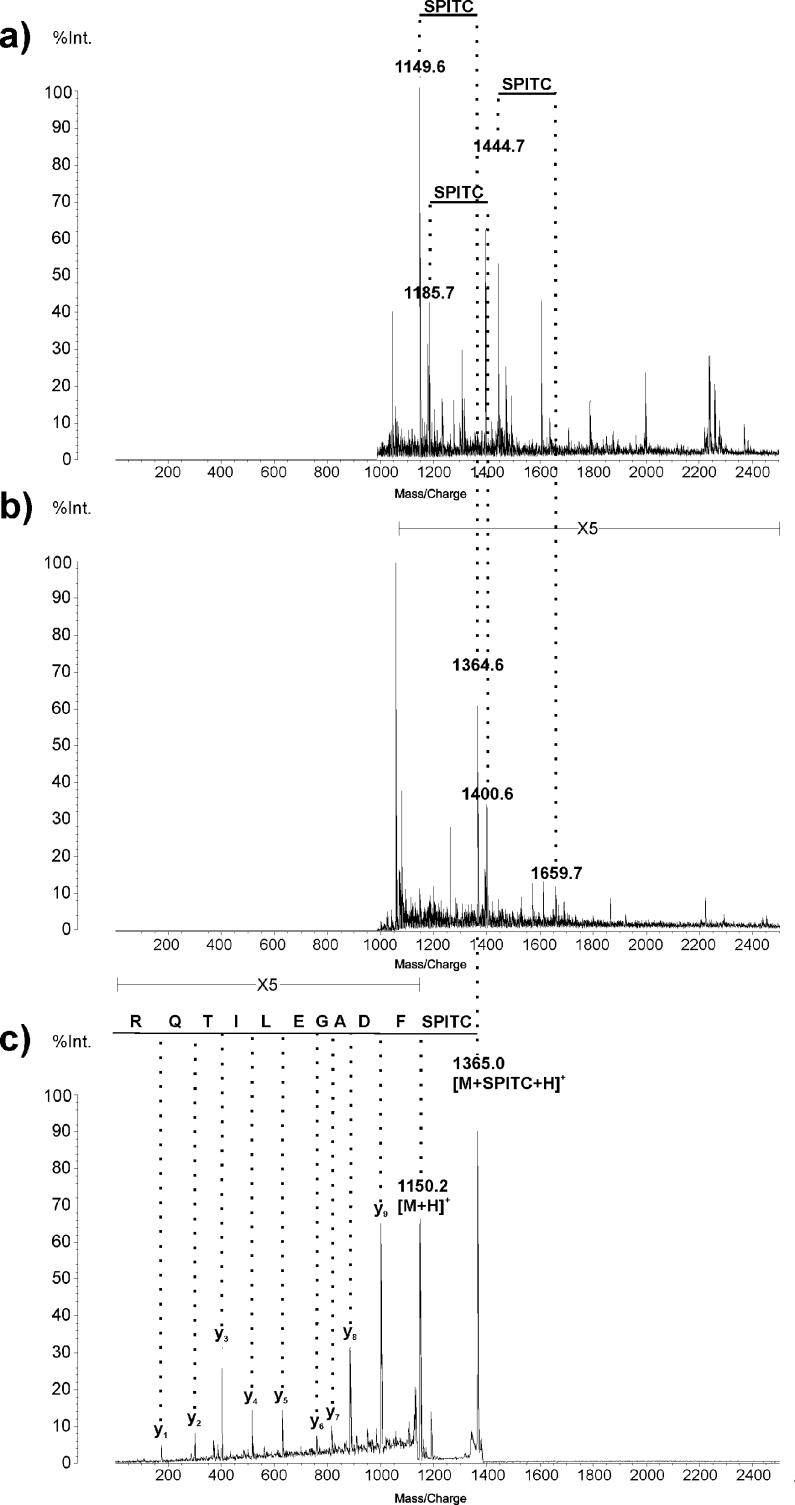
**MALDI-ToF MS spectra of *prohibitin***. The *peptide mass fingerprint *of prohibitin is shown before (a) and after (b) solid phase supported SPITC derivatisation as well as the PSD spectrum of the m/z value 1364.6 along with the corresponding y-series representing the primary sequence (c).

Improved protein identification by combined Mascot search of PMF and PSD spectra is shown exemplarily in Figure 1 for the spot C-5, human prohibitin (PHB). Database search of the underivatised PMF has not led to unambiguous protein identification. The best hits held protein scores between 55 and 57 at a threshold value for significance of 64. Among these, prohibitin is ranked third with a score of 56.

After SPITC derivatization, the ten most intensive m/z values of the derivatised PMF were selected by an ion gate for PSD fragmentation. The amino acid sequence of selected peptides can be distinguished considering the series of y-fragments. For the peptide of m/z 1365.0 the sequence FDAGELITQR (Figure 1c), for the peptide of m/z 1401.1 the sequence DLQNVNITLR (data not shown) and for the peptide of m/z 1160.3 the sequence IFTSI[GE]DYDER (data not shown) was confirmed.

Sequencing enables the mapping of a peptide to a certain protein within the confidence interval of its m/z value and increases its Mascot score. If the sequenced peptides do not represent homologue sections in the human proteome even one peptide sequence can be significant. In case of prohibitin each of the three peptides alone was sufficient for unambiguous protein identification. The combined search of PMF and PSD spectra of SPITC-derivatised peptides identifies spot C-5 as being human PHB with a Mascot Ion Score of 237 at a threshold for significance of 34.

### Enhanced sensitivity of SPITC derivatization on solid phase support

**Figure 2:**
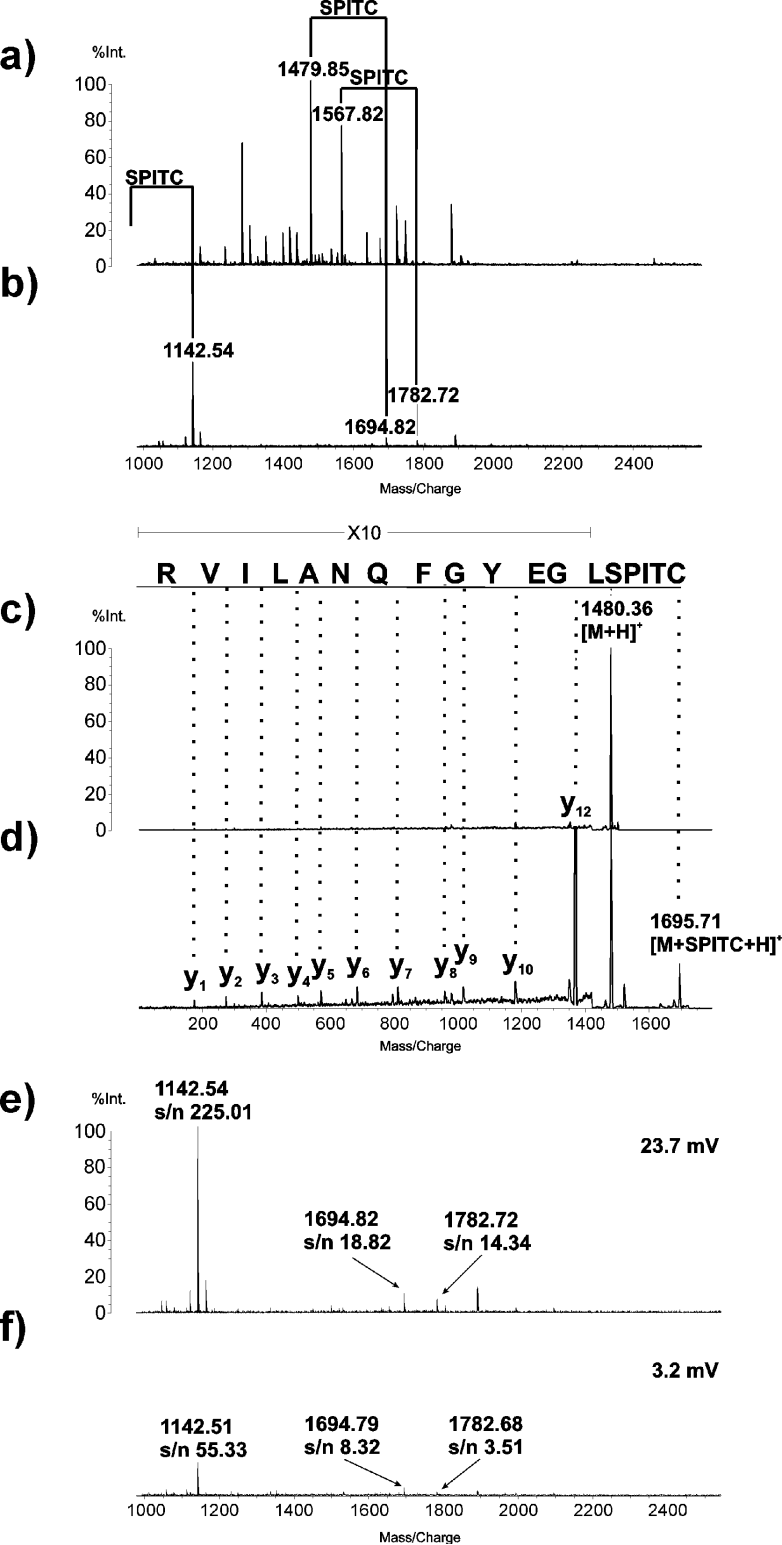
**MALDI-ToF MS spectra of *serum albumin (bos taurus)***. The pmf is faced before (a) and after (b) solid phase supported SPITC derivatization. The PSD spectra of m/z 1480,4 are shown before (c) and after (d) the derivatization along with the y-series. The pmfs of solid phase supported (e) and in solution (f) derivatization are opposed with the s/n ratios of identified peptides and the overall signal intensity charted.

The peptide mass fingerprint of tryptic digested protein standard *serum albumin (bovine) *before and after SPITC derivatization is shown in Figure 2. The intensity is normalised on m/z value of 1479.9 of underivatised PMF spectra. The SPITC derivatization introduces a mass shift of 215 Da to the peptides and has strongly decreased their intensities. Many peptides could no longer be detected after derivatization, which negatively affects the sensitivity of protein identification. The underivatised protein standard was unambiguously identified with a MascotScore of 129 and a significant threshold of 61, but could no longer be identified from the PMF after SPITC derivatization.

The influence of the N-terminal sulfonation on the quality of PSD spectra is shown exemplarily in Figure 2c and 2d for the m/z value of 1480.4 of 10 pmol tryptic digested BSA. The intensity is normalised on the precursor ion of the underivatised PSD spectra. Despite the reduction of the precursor ions' intensity of 95%, the signal to noise (s/n) ratio of the peaks in the PSD spectra as well as the frequency of fragmentation were clearly increased by the N-terminal sulfonation. The improved coverage of the precursor ion y-series raised the Mascot Ion Score of the peptide with the mass of 1480.4 Da from 10 to 88.

The sulfonation of primary amines was done in aqueous solution at slightly alkaline pH value (pH = 8.6). Purification of peptides was carried out with ZipTipμ-C18 columns according to the principle of reversed phase chromatography. Generally, SPITC derivatization of peptides can be performed in solution prior to column binding as well as solid phase supported. Both sulfonation methods were modified compared to the literature with respect to signal intensity in order to enable protein identification with maximum sensitivity ^15-16^. Exemplarily, the resulting PMF spectra for in-solution trypsinated BSA has been compared with the solid phase supported approach concerning signal intensity, which is shown in Figure 2e/f. The signal to noise ratios (s/n) of m/z values used for identification were about 2 to 4 times higher for peptides derivatised on solid phase support than for the ones sulfonated in solution. The overall signal of PMF spectra in the mass range between 1000 and 2500 m/z is about 7 times larger for the standard derivatised on C18 columns. The improved s/n ratio and the increased signal intensity of the solid phase supported approach in PSD mode enlarges the coverage of the precursor ions' y-series and simplifies the automation of peak picking. The Mascot Score of the database search of ten accumulated PSD spectra was 176 for the solid phase supported approach and 139 for in solution sulfonation at a threshold value for significance of 33.

### Comparative Proteome Analysis of VACV IHD-W infected HEK293 cells

**Figure 3:**
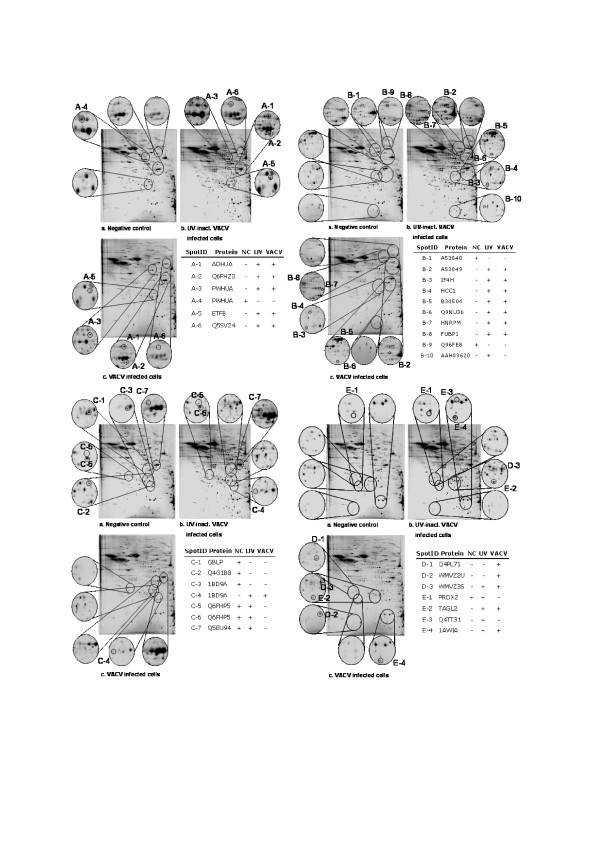
**Identified protein spots in 2D gels**. The spots of identified proteins are shown in the 2D gels of the negative control, HEK293 cells infected with VACV IHD-W and HEK293 incubated with UV inactivated VACV IHD-W concerning their classification. (+ identified, - not identified)

The comparative Proteome Analysis of infectious and UV-inactivated VACV IHD-W-infected HEK293 cells with non-infected control cells led to the identification of 24 modulated human proteins as well as 3 viral proteins. The proteins were identified via offline-coupling of 2D-SDS-PAGE (Figure 3) in the pI range of 4-9 and the mass range of 10-250 kDa with MALDI-PSD ToF MS and are classified according to their physiological role in the dynamic proteome. The five protein classes are proteins relevant for energy metabolism (A), proteins associated with gene expression and protein biosynthesis (B), proteins relevant for apoptosis (C), viral proteins (D) and other proteins (E). Proteins of the same class are numbered consecutively. The positions of the proteins identified in the 2DE gel are shown in different apertures in Figure 3. The molecular weight and pI values of these proteins are compared with the theoretical values in the SwissProt database (table 1). The entire protein denotations are listed in table 2.

**Table 1:** Molecular masses and pI values of identified proteins

*SpotID*	*Swiss Prot Accession #*	*Mascot Score*	*#* *peptides (PSD)*	*Sequence* *coverage [%]*	*theor. MW [kDa]*	*MW [kDa]*	*Δ MW [kDa]*	*theor. pI*	*pI*	*ΔpI*
A-1	P04075	45(35)	2	7	40	41	-1	8.3	7.5	0.8
A-2	Q6FHZ0	179(36)	2	8	36	34	2	8.9	7.6	1.3
A-3	P25705	132(35)	3	6	60	50	10	9.2	6.3	2.9
A-4	P25705	94(35)	3	5	60	49	11	9.2	5.8	3.4
A-5	P38117	86(36)	2	9	28	26	2	8.3	6.5	1.8
A-6	Q5SV24	117(35)	3	5	65	63	2	9.5	7.4	2.1
B-1	P41091	71(20)	1	2	52	52	0	8.7	6.4	2.3
B-2	P41091	44(35)	1	2	52	50	2	8.7	7.5	1.2
B-3	Q15056	95(39)	1	5	27	26	1	6.9	6.0	0.9
B-4	HCC1	57(36)	1	6	24	31	-7	6.1	6.1	0.0
B-5	P22626	259(35)	3	10	38	35	3	9.0	7.3	1.7
B-6	Q9NU36	48(33)	1	4	29	27	2	9.1	7.2	1.9
B-7	HNRPM	121(36)	4	6	78	66	12	8.9	6.8	2.1
B-8	Q96AE4	140(36)	3	5	68	73	-5	7.2	5.9	1.3
B-9	Q96FE8	37(34)	1	2	69	98	-29	9.4	5.6	3.8
B-10	O60925	75(36)	1	9	14	14	0	6.3	5.9	0.4
C-1	P63244	282(36)	4	13	36	31	5	7.6	6.1	1.5
C-2	Q4G1B8	117(42)	1	6	24	25	-1	8.6	6.4	2.2
C-3	1BD9A	71(36)	2	7	21	20	1	7.2	6.3	0.9
C-4	1BD9A	55(36)	1	7	21	21	0	7.2	6.4	0.8
C-5	Q6FHP5	237(34)	3	11	30	29	1	5.6	5.5	0.1
C-6	Q6FHP5	176(35)	3	11	30	28	2	5.6	5.5	0.1
C-7	Q5EU94	95(41)	1	4	33	37	-4	4.7	6.9	-2.2
D-1	Q4PL71	173(37)	2	11	22	24	-2	5.4	5.3	0.1
D-2	P20535	84(38)	2	15	13	14	-1	5.3	5.4	-0.1
D-3	P20642	66(38)	1	9	23	25	-3	5.5	6.3	-0.8
E-1	P32119	31(28)	1	5	22	21	1	5.7	5.5	0.2
E-2	P37802	158(35)	3	17	23	21	2	8.5	5.9	2.6
E-3	Q4TT31	37(35)	1	6	19	17	2	8.7	6.8	1.9
E-4	1AWIA	92(36)	1	10	15	14	1	8.5	6.7	1.8

**Full names of identified proteins:** Full names of identified proteins

*SpotID*	*NC*	*UV*	*VACV*	*Accession #*	*Protein*
A-1	-	+	+	P04075	Fructose-bisphosphate Aldolase A
A-2	-	+	+	Q6FHZ0	Malate dehydrogenase 2
A-3	-	+	+	P25705	H^+^-transporting two-sector ATPase alpha chain precursor
A-4	+	-	-	P25705	H^+^-transporting two-sector ATPase alpha chain precursor
A-5	-	+	+	P38117	Electron transfer flavoprotein subunit beta (Beta-ETF)
A-6	-	+	+	Q5SV24	ATPase family, AAA domain containing 3A
B-1	+	-	-	P41091	Translation initiation factor eIF-2 gamma chain
B-2	-	+	+	P41091	Translation initiation factor eIF-2 gamma chain
B-3	-	+	+	Q15056	Eukaryotic translation initiation factor 4H (eIF-4H)
B-4	-	+	+	HCC1	Nuclear protein Hcc-1 (CIP29)
B-5	-	+	+	P22626	Heterogeneous nuclear ribonucleoprotein B1
B-6	-	+	+	Q9NU36	Novel protein similar to small nuclear ribonucleoprotein polypeptide A
B-7	-	+	+	HNRPM	Heterogeneous nuclear ribonucleoprotein M (hnRNP M)
B-8	-	+	+	Q96AE4	Far upstream element-binding protein 1 (FUSE-binding protein 1)
B-9	+	-	-	Q96FE8	Ewing sarcoma breakpoint region 1, isoform EWS (EWSR1 protein)
B-10	-	+	-	O60925	Prefoldin subunit 1
C-1	+	-	-	P63244	Guanine nucleotide binding protein
C-2	+	-	-	Q4G1B8	PSMA8 protein
C-3	+	-	-	1BD9A	Phosphatidylethanolamine binding protein, chain A
C-4	-	+	+	1BD9A	Phosphatidylethanolamine binding protein, chain A
C-5	+	+	-	Q6FHP5	Prohibitin
C-6	+	+	-	Q6FHP5	Prohibitin
C-7	+	+	-	Q5EU94	Nucleophosmin
D-1	-	-	+	Q4PL71	Putative double-stranded RNA binding protein - vaccinia virus
D-2	-	-	+	P20535	14K cell fusion protein - vaccinia virus
D-3	-	+	+	P20642	major core protein P4a - 23kDa - vaccinia virus
E-1	-	+	+	P32119	Peroxiredoxin-2 (Natural killer cell-enhancing factor B)
E-2	+	+	-	P37802	Transgelin-2 (SM22-alpha homologue)
E-3	-	+	+	Q4TT31	Sorting nexin 3
E-4	-	+	+	1AWIA	Profilin, chain A

NC = negative control,

UV = UV inactivated VACV IHD-W infected HEK293,

VACV = VACV IHD-W infected HEK293

+ identified, - not identified

## Discussion

### Protein identification via MALDI-PSD-ToF MS spectra of on solid phase support SPITC derivatized peptides

The recording of PSD spectra from m/z values of peptide mass fingerprints provides information about the peptides' primary structure and enables the unambiguous identification of proteins from few peptides with significant Mascot ion scores by an analysis of fragmentation patterns. The difficulties of protein identification with MALDI-PSD-ToF MS spectra are low s/n ratios of fragments, incomplete sequence coverage and complex fragmentation patterns as a cause of random charge stabilisation. The protonation of peptides during the MALDI process is mediated by carboxyl groups of the matrix and mainly stabilised at primary amine groups of the N-termini and the Lysine residues. The stabilised charge induces different fragmentations of the same ion. The derivatization of peptides with SPITC binds a negatively charged sulfonic acid to the peptides' N-terminus [13]. This negative charge neutralises the protonation of C-terminal fragments which are no longer detectable in mass spectrometry. Primarily, the PSD spectra of SPITC-derivatised peptides show fragments of the y-series, whose appearance among the N-terminal fragments is promoted by the large electron density around the peptide bond. The derivatization with SPITC enables the detection of complete y-series with good s/n ratios and offers the elucidation of the peptides' primary sequence. The unambiguous mapping of peptides with completely known primary sequences to proteins registered in databases is remarkably simplified and is expressed in increased Mascot scores [14]. However, the application of SPITC derivatization for protein identification from complex mixtures is limited by the reduction of the peptides' signal intensities following N-terminal sulfonation. This degradation of sensitivity could be decreased by an optimization of the reaction conditions. For increased sensitivity the N-terminal sulfonation of peptides bound on C18 solid phase support is preferable compared to an in solution approach since signal intensity is increased and quality of PSD spectra is raised.

### Comparative Proteome Analysis of VACV IHD-W infected HEK293 cells

The comparison of the proteome analysis of VACV IHD-W-infected HEK293 cells with non-infected control cells 9 h post infection has enabled the identification of 24 human proteins, whose expression has been regulated by infection, as well as of 3 viral proteins. Since the genome of VACV IHD-W is not available, the identification of viral proteins occurred via sequence homologies of the VACV strains *Western Reserve *and *Copenhagen*. Afterwards peptide sequences were derived from spectra of 13 spots representing proteins regulated in their expression, whose database search gained no result. Single-base substitutions among different VACV strains, which led to amino acid replacement, might have resulted in different peptide mass fingerprints. PSD spectra are in correlation to the confidence interval of the m/z values of the precursor ion. Therefore, protein identification via Mascot Search is disabled by mass shifts of precursor ions resulting from an amino acid replacement. De novo sequencing of viral proteins could not be assured because of the polyproteonality of 2D-gel spots. The unambiguously identified differences in the expression profile of HEK 293 cells resulting from VACV IHD-W infection are discussed in the following sections.

### Proteins relevant for energy metabolism

The infection of HEK293 cells with active and inactivated Vaccinia Virus IHD-W significantly affects the cells' energy metabolism. The expression of *fructose-bisphosphate aldolase A (A-1)*, which acts as a central enzyme in the glycolysis by catalysing the retro-aldol cleavage of fructose-1,6-bisphosphate into dihydroxyacetone phosphate and glycerinaldehyde, is increased by both types of infection [15]. Also the enhanced expression of the enzyme *malate dehydrogenase (A-2) *is directly associated with a boost in the energy metabolism, which serves the viruses' need for energy for DNA replication. This enzyme is an integral part of the citric acid cycle by catalysing the oxidation of malate to fumarate [16]. The resulting reduction equivalent NADH/H^+ ^transfers electrons to the electron transport chain which builds up a proton gradient at the inner mitochondrial membrane, resulting in ATP-synthesis catalysed by the H*^+^-transporting two-sector ATPase (A-3, A-4) *[17]. A modification of the α-chain precursor of the *H^+^-transporting two-sector ATPase (A-3, A-4)*, resulting in spot migration, is triggered by both types of infection and has been detected as well as an overexpression of the mtDNA stabilising *ATPase family AAA domain containing 3A (A-6) *protein, whose existence has just been evidenced at transcript level [18]. Another energy metabolism-related protein whose expression is up-regulated by the VACV IHD-W infection is the *βsubunit of the electron transfer flavoprotein (A-5) *which acts as a specific electron acceptor for several dehydrogenases, e.g. *malate dehydrogenase (A-2)*, and transfers them to the respiratory chain [19].

All changes detected in the human proteome profile caused by the infection which are related to energy metabolism indicate an enhancement of the metabolic rate of the glycolysis as well as of the oxidative phosphorylation in order to fulfil the viruses' energy need for replication.

### Proteins associated with gene expression and protein biosynthesis

The expression of the *eukaryotic translation initiation factor 4H (eIF-4H) (B-3) *is up-regulated by infectious and inactivated VACV IHD-W. *The protein eIF-4H *stimulates protein biosynthesis, ATP hydrolysis and helicase activity of eIF-4A. Enzymatic investigations show that the affinity of eIF4A to RNA is increased two-fold and helicase activity four-fold by an interaction with eIF-4H [20]. The regulation of *eIF-4H *may contribute to an enhancement in protein biosynthesis which is required by the virus' need for protein expression.

Further on an increased expression of *nuclear protein Hcc-1 (CIP29) (B-4) *is denoted in both types of infected HEK293 cells. This protein possesses a binding domain for ss and dsDNA and is postulated to be part of the ribonuclein complex. Interactions with the RNA-helicases DDX39 and BAT 1 are verified which proves the influence of Hcc-1 on the transcription of DNA. The overexpression of Hcc-1 in HEK293 cells is known to decrease the cells' growth rate [21,22].

Several members of the spliceosome, *heterogeneous nuclear ribonucleoprotein B1 (B-5), novel protein similar to small nuclear ribonucleo-protein polypeptide A (B-6), heterogeneous nuclear ribonucleoprotein M (B-7)*, are modulated in both types of VACV IHD-W-infected HEK2093 cells. This heterogeneous protein complex splices introns of the hnRNA. Since the poxvirus genome has no introns and splicing of mRNA is therefore obsolete, viral effectors may control the cells' protein biosynthesis by interfering with the cellular hnRNA processing [23,24].

*Far upstream element-binding protein 1 (FUBP1) (B-8) *stimulates the expression of transcription factor c-myc by binding onto far upstream element (FUSE) upstream of the c-myc promoter [25]. The protein is a known link between the apoptosis cascade and the c-myc oncogene, it further possesses helicase activity for dsDNA. Experiments with transfected cells show that a high FUBP1 expression increases the expression level of c-myc and in this way protects the cell from apoptosis, while caspase-mediated cleavage of FUBP1 induces apoptosis [26]. Both infectious and inactivated VACV IHD-W enhance the expression of FUBP1.

*Ewing sarcoma breakpoint region 1 (B-9) *is a multifunctional protein with essential functions in gene expression, signal transduction, and mRNA transport and processing. Mutations in the protein-expressing gene lead to the development of Ewing sarcomas and other tumours. The protein expression is suppressed after an infection with UV-inactivated as well as with active viruses [27].

*Prefoldin subunit 1 (B-10) *is part of a heterohexamer chaperon (prefoldin) which binds cytosolic chaperonin (c.CPN) and transports target proteins to the hexamer. It is also involved in the folding of nascent peptides. Gene deletions of prefoldin result in a dysfunction of the actin-tubulin cytoskeleton [28]. Prefoldin *subunit 1 *is exclusively identified in cells infected with UV-inactivated VACV.

The described alterations caused by the infection are results of the fact that the virus is taking control of the cells' gene expression as well as the protein biosynthesis and so aims for effective expression of viral proteins and the control of the cellular response.

### Proteins relevant for apoptosis

Several viruses in general and poxviruses in particular are known to modulate the hosts' apoptosis pathways in order to avoid the antiviral defence mechanism at a cellular level [29]. This results in an altered expression profile of apoptosis-relevant genes caused by viral gene products. During VACV transcription double-stranded RNA (dsRNA) is synthesised, since the virus possesses overlapping genes on both strands of its DNA. The presence of dsRNA activates the dsRNA-dependent serine/threonine kinase (PKR) which phosphorylates the α-subunit of the translation initiation factor eIF-2 which is essential for protein biosynthesis. This phosphorylation inhibits the translation of mRNA and thereby induces apoptosis [30-33]. An altered modification on the γ-subunit of the translation initiation factor eIF-2 (B-1, B-2) caused by the infection was detected by a spot migration in the 2-DE gel the relevance of which has not yet been determined in the literature. The dsRNA-dependent PKR is usually inhibited by *nucleophosmin 1 (C-7) *which is ubiquitously expressed in human cells and translocates between nucleus and cytoplasm [34]. The downregulation of *nucleophosmin 1 (C-7)*, which can not be detected in cells infected with active VACV IHD-W, suggests that it is a cellular response to the infection for the purpose of anti-viral defence. This immune response is undercut by the product of the VACV E3L gene, the *putative double-stranded RNA binding protein (D-1) *which binds dsRNA and inhibits apoptosis induction by preventing PKR from phosporylating the γ-subunit of the translation initiation factor eIF-2 [35,36]. The E3L gene product has been identified in HEK 293 cells infected with active VACV IHD-W, which is in correlation to proteome analysis of VACV virions that indicate that the *putative double-stranded RNA binding protein (D-1) *is not present in the virion but is expressed in the early replication phase.

The present proteome analysis has led to the identification of several further apoptosis-relevant proteins. The expression of two isoforms of *prohibitin (C-5, C-6) *is decreased after infection with active VACV IHD-W. *Prohibitin *regulates cell division, inhibits DNA synthesis and sensibilises cells for apoptosis by destabilising the mitochondrial membrane [37]. Beyond that, *prohibitin *enhances the transcription of p53 [38].

The infection of HEK293 cells with active and inactivated VACV IHD-W inhibits the expression of the protein *Guanine nucleotide-binding protein subunit beta 2-like 1 (C-1) *which is an acceptor for activated protein kinase C (PKC). PKC is then bound to the cytoskeleton and conveyed to its target proteins, the MARCKS proteins. The receptor is also engaged in the regulation of the SRC kinase which can inhibit apoptosis by a phosphorylation of caspase-8 [39,40].

The reduced expression of the *proteasome subunit alpha type-7-like (C-2*) can be seen as a further cellular defence strategy. The *proteasome subunit alpha type-7-like (C-2) *is part of the proteasome complex which cleaves peptides proteolytically at the amino acids Arg, Phe, Tyr, Leu and Glu [41]. The inhibition of the proteasome in tumour cell lines induces apoptosis.

The *phosphatidylethanolamine-binding protein (C-3, C-4) *is modified by an infection with active and inactivated VACV IHD-W, which results in an altered pI value. The protein binds to phosphatidylethanolamine which is presented at the surface of apoptotic cells, inhibits the Raf-kinase, prevents the activation of the MAP-Kinase-cascade and the antiapoptotic transcription factor NFκ-B [42,43]. It is to be postulated that the protein modification is associated with an altered physiological activity.

### Viral proteins

In this comparative proteome analysis the *14 kDa cell fusion protein (D-2) *is identified in HEK293 cells infected with active VACV IHD-W. Its presence is predicted according to Swissprot [44]. The variola homologue of the A27L gene is known to play an import role in virus penetration by fusing the outermost of the Golgi-derived membranes enveloping the virus with the cells' plasma membrane.

As expected, the *major core protein P4a (D-3) *of Vaccinia virions is further identified in both infected cell culture approaches [45].

### Other proteins

*Peroxiredoxin-2 (E-1) *is an antioxidative enzyme which reduces hydrogen- and alkyl-peroxides and supports the antiviral activity of CD8(+) T-cells [46]. In contrast to the negative control, protein expression is enhanced by an infection with active and UV-inactivated viruses.

*Transgelin-2 (SM22-alpha homologue) (E-2) *has not been functionally characterised yet. The protein possesses an actin-binding domain and is overexpressed in cells infected with both infectious and inactivated VACV IHD-W. It seems as if human transgelin is packed into Vaccinia virions and transferred from cell to cell for an unknown reason [47]. It can be hypothesised that it may serve as an anchor for the virus to move along the cytoskeleton.

*Sorting nexin *3 *(E-3) *has a binding domain (PX domain) for phospholipids and transports proteins within cells from organelles to membranes [48]. The protein expression is enhanced after infection of HEK293 cells with active and UV-inactivated VACV IHD-W.

*Profilin (E-4) *binds actin and participates in the build-up of the cytoskeleton. As a response of extracellular signals, high concentrations of *profilin *inhibit the Actin-polymerization which is enhanced by low *profilin *concentrations [49]. Both types of infection increase expression of human profilin, while VACV possesses a *profilin *homologue by itself.

## Conclusions

Chemical assisted fragmentation (Caf) of peptides by N-terminally sulfonation with SPITC improves confidence in protein identification from PSD spectra. ZipTipμ-C18 columns solid phase-supported SPITC derivatization is preferable compared to an in-solution approach, since s/n ratios as well as signal intensities are increased, and so the sensitivity for recording high quality PSD spectra is enhanced. Proteome analysis of VACV IHD-W-infected HEK293 cells in the late replication phase shows the up-regulation of the energy metabolism and alteration of gene expression regulation. Further on the modulation of apoptosis which counteracts the cellular anti-viral response is demonstrated by the regulation of several proteins from different signalling pathways. Interestingly the effects of UV-inactivated viruses and infectious viruses on the hosts' proteome are quite similar. There is no difference between the identified alterations of the energy metabolism and just one differentially expressed protein associated with gene expression and protein biosynthesis (B-10). Therefore the up-regulation of the energy metabolism and alterations in the regulation of the cellular gene expression are results of virus entry and virions' proteins. However, the modulation of apoptosis is clearly affected by effects that correlate with infectious viruses. The absence of *nucleophosmin *in cells infected with infectious viruses, for example, may be the response on viral dsRNA resulting from transcription of viral DNA, as the competing apoptosis modulator *putative double-stranded RNA binding protein (D-1) *is not a part of the virion.

In part, the identified proteins have not yet been described in the context of poxvirus infections and need to be further characterised using virological methods to identify their meaning for apoptosis modulation and pathogenesis.

## Methods

### Chemicals and equipment

All of the electrophoresis apparatus (IPGphor II, Multiphor II chamber Electrophorese, EPS 3500 power supply) were purchased from Amersham Pharmacia Biotech (Uppsala, Sweden). IPG DryStrips and Pharmalyte pH 3-10 were obtained from GE Healthcare (Chalfont St Giles, Great Britain). Acrylamide, bis-acrylamide, iodoacetamide (IAA), thiourea, dithiothreitol (DTT), 3-[(3-Cholamidopropyl)-dimethylammonio]-1-propane sulfonate (CHAPS), a-cyano-4-hydroxycinnamic acid (CHCA), acetonitrile (ACN), trifluoroacetic acid (TFA), 4-sulfophenyl isothiocyanate (SPITC), biammonium citrate, bovine serum albumin (BSA), sodium dodecyl sulfate (SDS), Trypsin Profile IDG Kit and the ProteoSilver Plus Silver Stain Kit were bought from Sigma-Aldrich (Taufkirchen, Germany). Tris (hydroxymethyl) aminomethane (TRIS) and solvents used (dichloromethane, ethanol, isopropanol) were supplied in analytical form by Merck (Darmstadt, Germany). Dulbecco's Modified Eagle Medium (DMEM) as well as foetal bovine serum (FBS) were purchased from Gibco (Eggenstein, Germany). TRIzol^® ^Reagent was purchased from Invitrogen (Carlsbad, USA). Ultrapure water was generated by a water purification system and used throughout for standard and sample preparation.

### Cell Culture and Proteome Extraction

HEK 293 cells (ATCC-CRL-1573™) were cultured in three 150 cm^2 ^cell culture flasks with 25 ml culture media (DMEM containing 5% FBS [v/v] and 4 mM L-glutamine) up to a density of 2.3 × 10^7 ^cells/flask at 37°C and 5% CO_2 _in an incubator. Cells in one flask were infected with VACV IHD-W (ATCC-VR-1441™) and in the other one with UV-inactivated VACV IHD-W with a multiplicity of infection (MOI) of 5. Cells in the third flask were cultured as a negative control. 1 h post infection the infection media (11.5 × 10^7 ^plaque forming units [PFU] in 10 ml culture media/flask) was exchanged for fresh 25 ml culture media. Proteomes of all three cell culture approaches were purified 9 h post infection by use of TRIzol^® ^Reagent (Invitrogen) according to the manufacturer's instructions. Dried proteome pellets were stored at -20°C until further use.

### UV inactivation of VACV IHD-W

Virus stock solution containing 11.5 × 10^7 ^PFU was centrifuged for 1 h at 21,000 × *g *and 4°C. The virus pellet was resuspended in 1 ml phosphate buffered saline (PBS), transferred into a 6-well plate and inactivated by UV irradiation of 0.05 W/m^2 ^in a StrataLinker 2400. Success of inactivation was checked by immunofluorescence staining of infected cells.

### Two-Dimensional Polyacrylamide Gel Electrophoresis: 2D-SDS-PAGE

The two-dimensional polyacrylamide gel electrophoresis with immobilised pH gradient was performed according to the procedure by Görg et al [50]. Initially, 100 μg dried protein was solubilised in 400 μl rehydration solution (8 M urea, 2 M thiourea, 16 mM Chaps, 20 mM DDT, 0.5 vol.-% Pharmalyte 3-10, 10 μg bromphenol blue) and loaded onto isoelectric focusing strips with a linear gradient (pH 3-10, L = 18 cm) for 12 h. The rehydration occurred up to a thickness of 0.5 mm for 12 h passively and for 2 h actively, before the isoelectric focusing was done with the following parameters in 8 steps: S1: 100 V 14 h, S2: 100 V to 1800 V 30 min, S3: 1800 V 1 h, S4: 1800 V to 6000 V 40 min, S5: 6000 V 2 h, S6: 6000 V to 8000 V 15 min, S7: 8000 V 3 h, S8: 8000 V 5 h. For equilibration, the proteins were reduced for 15 min at 60°C with solution A (10 vol.-% 0.5 M Tris-HCl, pH 6.8, 6 M urea, 30 vol.-% glycerol, 140 mM SDS, 20 mM DDT) and then alkylated with solution B (10 vol.-% 0.5 M Tris-HCl, pH 6.8, 6 M urea, 30 vol.-% glycerol, 140 mM SDS, 240 mM iodoacetamid, bromphenol blue) for 15 min at 25°C in the dark. In the second dimension the proteins were separated in a 12-%-polyacrylamide gel (250 × 200 × 1 mm^3^) at 4 W, 600 V and 30 mA for 14 h at 12°C in a horizontal system. The size marker was RotiMark^® ^10-150 kDa (Roth, Karlsruhe, Germany) and a Tris-glycine buffered system according to Laemmli (1970) was used [51]. After electrophoresis, the proteins were silver stained with the ProteoSilver Plus Silver Stain Kit (Sigma-Aldrich) according to the manufacturer's instructions.

### Gel image analysis

Differences in the protein expression profile of the 2-D gels were identified by use of the image analysis software Melanie 7.0 (Swiss Institute for Bioinformatics). A quantitative analysis was disclaimed since silver staining is not an end point method. A match set was created from scanned gel images in order to compare protein spots across gels. The gel containing the best resolution and most spots was chosen as the master gel. Landmark spots were defined and manual editing of the matching procedure was performed to improve the automated matching results. Finally, the mismatched spots representing differences in protein expression were manually checked and selected for Mass Spectrometric analysis.

### In-gel trypsination

Differences in the protein expression profile of the 2-D gels were identified by use of the image analysis software Melanie 7.0 (Swiss Institute for Bioinformatics, Lausanne, Switzerland, http://www.expasy.org/melanie). A quantitative analysis was disclaimed since silver staining is not an end-point method. Spots representing differences in protein expression were excised as dice with 1 mm edge length each, which were destained with the ProteoSilver™ Plus Silver Stain Kit (Sigma-Aldrich) according to the manufacturer's instructions. Gel dice were then dried in a SpeedVac after dehydration in 100 μl ACN. The dried dice were swollen in 10 μl reaction buffer (25 mM ammonium bicarbonate [NH_4_HCO_3_] pH 8.0) containing 500 ng trypsin (Trypsin Profile IGD Kit, Sigma-Aldrich) for 15 min at 4°C, before another 40 μl reaction buffer was added. The tryptic digest was incubated overnight at 37°C. The proteolytic peptide containing supernatant was transferred into a new reaction tube. Peptides remaining in the gel dice were extracted by adding 20 μl 1:1 ACN/0.1%TFA for 30 min. Pooled peptides were evaporated to dryness in a SpeedVac for further analysis.

### In-solution trypsination of serum albumin *(bos taurus)*

Reduction of disulfide bonds was done by addition of 2 μl 2 M dithiotreitol (DTT) to 200 μl 0.1 μg/μl BSA in 25 mM NH_4_HCO_3 _pH 8.0 at 60°C for 30 min. Subsequently free thiol groups were carboxyamidomethylated by adding 6 μl 1 M IAA at 25°C for 30 min in the dark. Excessive iodoacetamide (IAA) was converted with 1 μl 2 M DTT at 60°C for 15 min before the proteolytic digest was induced by addition of 1 μg trypsin (1:20 w/w) and incubated for 16 h at 37°C. For SPITC derivatization the desired amount of protein was transferred to a microcentrifuge tube and evaporated to dryness in a SpeedVac.

### N-terminal peptide derivatization with 4-sulfophenyl isothiocyanate on ZipTipμ-C18 columns

Dried peptides were resolved in 5 μl 0.1% TFA, 0.5 μl of which were mixed with 0.5 μl matrix (10 mg α-Cyano-4-hydroxycinnamicacid [CHCA] in 1 ml ACN/0.1% TFA [1:1 v/v]) on a stainless steel carrier and were air-dried. Prior to the recording of peptide mass fingerprints, the spots were washed with 1.5 μl 10 mM biammonium citrate three times. The remaining peptide solution was bound to a ZipTipμ-C18 (Millipore) pipette tip which was wetted with 10 μl ACN/0.1% TFA (1:1 v/v) and washed twice with 10 μl 0.1% TFA. Subsequently the ZipTipμ-C18 was loaded with 10 μl 5 mg/ml 4-Sulfophenyl isothiocyanate (SPITC) in 20 mM NaHCO_3 _pH 8.6 and incubated at 55°C for 1 h in a circulating air oven. Derivatised peptides were washed five times with 0.1% TFA and three times with 10 mM biammonium citrate prior to elution with 1 μl ACN/0.1% TFA (1:1 v/v) directly onto the MALDI target. 0.5 μl matrix (10 mg CHCA in 1 ml ACN/0.1% TFA [1:1 v/v]) was added and the spot was air-dried.

### N-terminal peptide derivatization with 4-sulfophenyl isothiocyanate in aqueous solution

Dried peptides were resolved in 10 μl derivatization solution (10 mg/ml 4-Sulfophenyl isothiocyanate [SPITC] in 20 mM NaHCO_3 _pH 8.6) and incubated at 60°C for 30 min. Purification, elution and co-crystallization of derivatised peptides was done by use of ZipTipμ-C18 (Millipore) pipette tips as described above.

### MALDI-ToF curved field reflectron (CFR)

The mass spectrometric investigations have been carried out using an AXIMA-CFR^PLUS ^(Kratos Analytical, Manchester, UK). Peptide mass fingerprints were recorded in positive ion reflectron mode with a setting of the laser intensity of 2.2 μJ and a maximum laser-repetition rate of 5.0 Hz at a pulse extraction of 1500 m/z (Delay-time). Each recorded spectrum was the sum range of 200 profiles, accumulated from five laser shots. Ten m/z values greater than 1200 were automatically selected with descending intensity by the Launchpad Software (Kratos Analytical) for post source decay (PSD) measurement. 100 profiles accumulated from ten laser shots were accumulated for one PSD spectrum which was recorded in positive ion reflectron mode with a laser intensity of 4.0 μJ and a maximal laser-repetition rate of 5.0 Hz at a pulse extraction of 1500 m/z (delay-time). An external 5-points calibration was performed by using appropriate peptide standard (*calibration Mix [Proteomix] 500-3500 Da *[LaserBio Labs]) prior to each measurement. A combined data file of m/z values of the PMF and PSD spectra was sent to the ExPAsy Server (Swiss Institute for Bioinformatics) and synchronised with the SwissProt database for protein identification. Peaks from the raw data were picked using MascotDistiller (Matrix Science, Boston, USA) with a minimal signal/noise of 3 and a peak width of 0.3 m/z values were annotated with a mass tolerance of +/-0.3 Da in MS^1 ^and +/-0.7 Da in PSD mode. Carbamidomethylated cystein residues and SPITC derivatised N-termini were set as fixed modifications, oxidised methionine residues as a variable modification.

## Competing interests

The authors declare that they have no competing interests.

## Authors' contributions

JD carried out the two-dimensional gel electrophoresis, image analysis as well as the mass spectrometric measurements. SB established the method for fragmentation of SPITC derivatized peptides and executed the database searches for protein identification. The manuscript was drafted by SB and JD in equal parts. The cell culture part of this study was performed by DB, whereas KD supported the image analysis and database searches. RB financed the analytical part of the study. HL coordinated the study and gave relevant contributions to the technical background. AN conceived the study, and participated in its design. All authors read and approved the final manuscript.

## References

[B1] Morens DM Folkers GK Fauci AS The challenge of emerging and re-emerging infectious diseases Nature 2004 430 242 249 10.1038/nature02759 15241422 PMC7094993

[B2] Finlay BB See RH Brunham RC Rapid response research to emerging infectious diseases: lessons from SARS Nature 2004 2 602 607 10.1038/nrmicro930 PMC7097457 15197395

[B3] Di Giulio DB Eckburg PB Human monkeypox: an emerging zoonosis Lancet Infect Dis 2004 4 15 25 10.1016/S1473-3099(03)00856-9 14720564 PMC9628772

[B4] Vorou RM Papavassiliou VG Pierroutsakos IN Cowpox virus infection: an emerging health threat Curr Opin Infect Dis 2008 21 153 156 10.1097/QCO.0b013e3282f44c74 18317038

[B5] Kortepeter MG Parker GW Potential biological weapons threats Emerg Infect Dis 1999 5 523 527 10.3201/eid0504.990411 10458957 PMC2627749

[B6] Blasco R Sisler JR Moss B Dissociation of progeny vaccinia virus from the cell membrane is regulated by a viral envelope glycoprotein: effect of a point mutation in the lectin homology domain of the A34R gene J Virol 1993 67 3319 3325 8497053 10.1128/jvi.67.6.3319-3325.1993 PMC237674

[B7] Chung CS Hsiao JC Chang YS Chang W A27L protein mediates vaccinia virus interaction with cell surface heparan sulfate J Virol 1998 72 1577 1585 9445060 10.1128/jvi.72.2.1577-1585.1998 PMC124638

[B8] Hsiao JC Chung CS Chang W Cell surface proteoglycans are necessary for A27L protein-mediated cell fusion: identification of the N-terminal region of A27L protein as the glycosaminoglycan-binding domain J Virol 1998 72 8374 8379 9733888 10.1128/jvi.72.10.8374-8379.1998 PMC110218

[B9] Hsiao JC Chung CS Chang W Vaccinia virus envelope D8L protein binds to cell surface chondroitin sulfate and mediates the adsorption of intracellular mature virions to cells J Virol 1999 73 8750 8761 10482629 10.1128/jvi.73.10.8750-8761.1999 PMC112896

[B10] Lin CL Chung CS Heine HG Chang W Vaccinia virus envelope H3L protein binds to cell surface heparan sulfate and is important for intracellular mature virion morphogenesis and virus infection in vitro and in vivo J Virol 2000 74 3353 3365 10.1128/JVI.74.7.3353-3365.2000 10708453 PMC111837

[B11] Yoder JD Chen TS Gagnier CR Vemulapalli S Maier CS Hruby DE Pox proteomics: mass spectrometry analysis and identification of Vaccinia virion proteins Virology J 2006 3 10 10.1186/1743-422X-3-10 PMC1540416 16509968

[B12] Zhang L Villa NY Rahman MM Smallwood S Shattuck D Neff C Dufford M Lanchbury JS Labaer J McFadden G Analysis of vaccinia virus-host protein-protein interactions: validations of yeast two-hybrid screenings J Proteome Res 2009 8 9 4311 4318 10.1021/pr900491n 19637933 PMC2738428

[B13] Chen P Nie S Mi W Wang XC Liang SP De novo sequencing of tryptic peptides sulfonated by 4-sulfophenyl isothiocyanate for unambiguous protein identification using post-source decay matrix-assisted laser desorption/ionization mass spectrometry Rapid Commun Mass Spectrom 2004 18 191 198 10.1002/rcm.1280 14745769

[B14] León IR Neves- Ferreira AG Valente RH Mota EM Lenzi HL Perales J Improved protein identification efficiency by mass spectrometry using N-terminal chemical derivatization of peptides from *Angiostrongylus costaricensis*, a nematode with unknown genome J Mass Spectrom 2007 42 1363 1374 10.1002/jms.1324 17902111

[B15] Sibley JA Lehninger AL Determination of aldolase in animal tissues J Biol Chem 1949 177 2 859 872 18110462

[B16] Witt I Kronau R Holzer H The Isoenzymes of *malate dehydrogenase *and their regulation in *Saccharomyces cerevisiae* Biochim Biophys Acta 1966 128 1 63 73 5972369

[B17] Champagne E Martinez LO Collet X Barbaras R Ecto-F1Fo ATP synthase/F1 ATPase: metabolic and immunological functions Curr Opin Lipido 2006 17 279 284 10.1097/01.mol.0000226120.27931.76 16680033

[B18] Martin S Nucleotide Sequence. *Submitted (JAN-2009) to the EMBL/GenBank/DDBJ databases *2009, Cited for: NUCLEOTIDE SEQUENCE

[B19] Colombo I Finocchiaro G Garavaglia B Garbuglio N Yamaguchi S Frerman F Berra B Didonato S Mutations and polymorphisms of the gene encoding the beta-subunit of the electron transfer flavoprotein in three patients with glutaric acidemia type II Hum Mol Genet 1994 3 429 435 10.1093/hmg/3.3.429 7912128

[B20] Richter NJ Rogers GW Hensold JO Merrick WC Further Biochemical and Kinetic Characterization of Human Eukaryotic Initiation Factor 4H J Biol Chem 1999 274 35415 35424 10.1074/jbc.274.50.35415 10585411

[B21] Leaw CL Ren EC Choong ML Hcc-1 is a novel component of the nuclear matrix with growth inhibitory function Cell Mol Life Sci 2004 61 17 2264 2273 15338056 10.1007/s00018-004-4205-x PMC11138947

[B22] Fukuda S Pelus LM Growth inhibitory effect of Hcc-1/CIP29 is associated with induction of apoptosis, not just with G2/M arrest Cell Mol Life Sci 2005 62 13 1526 1527 10.1007/s00018-005-5093-4 15924260 PMC11139076

[B23] Kozu T Heinrich B Schaefer KP Structure and expression of the gene (HNRPA2B1) encoding the human hnRNP protein A2/B1 Genomics 1995 25 365 371 10.1016/0888-7543(95)80035-K 7789969

[B24] Jurica MS Licklider LJ Gygi SP Grigorieff N Moore MJ Purification and characterization of native spliceosomes suitable for three-dimensional structural analysis RNA 2002 8 426 439 10.1017/S1355838202021088 11991638 PMC1370266

[B25] Duncan R Bazar L Michelotti G Tomonaga T Krutzsch H Avigan M Levens D A sequence-specific, single-strand binding protein activates the far upstream element of c-myc and defines a new DNA-binding motif Genes Dev 1994 8 465 480 10.1101/gad.8.4.465 8125259

[B26] Jang M Park BC Kang S Chi SW Cho S Chung SJ Lee SC Bae KH Park SG Far upstream element-binding protein-1, a novel caspase substrate, acts as a cross-talker between apoptosis and the c-myc oncogene Oncogene 2009 28 12 1529 1536 10.1038/onc.2009.11 19219071

[B27] Embree LJ Azuma M Hickstein DD Ewing Sarcoma Fusion Protein EWSR1/FLI1 Interacts with EWSR1 Leading to Mitotic Defects in Zebrafish Embryos and Human Cell Lines Cancer Res 2009 69 10 4363 4371 10.1158/0008-5472.CAN-08-3229 19417137 PMC2766243

[B28] Vainberg IE Lewis SA Rommelaere H Ampe C Vandekerckhove J Klein HL Cowan NJ Prefoldin, a chaperone that delivers unfolded proteins to cytosolic chaperonin Cell 1998 93 863 873 10.1016/S0092-8674(00)81446-4 9630229

[B29] O'Brien V Viruses and apoptosis J Gen Virol 1998 79 1833 1845 9714231 10.1099/0022-1317-79-8-1833

[B30] Rice AP Interferon mediated, *double-stranded RNA-dependent proteine kinase *is inhibited in extracts from vaccinia virus-infected cells J Virol 1984 50 229 236 6699945 10.1128/jvi.50.1.229-236.1984 PMC255603

[B31] García MA Meurs EF Esteban M The *dsRNA proteine kinase PKR*: virus and cell control Biochimie 2007 89 6-7 799 811 10.1016/j.biochi.2007.03.001 17451862

[B32] Gil J Esteban M Induction of apoptosis by the *dsRNA-dependent protein kinase (PKR)*: mechanism of action Apoptosis 2000 5 2 107 114 10.1023/A:1009664109241 11232238

[B33] Whitaker-Dowling P Youngner JS Characterization of a specific kinase inhibitory factor produced by vaccinia virus which inhibits the *interferon *induced proteine kinase Virology 1984 137 171 181 10.1016/0042-6822(84)90020-5 6474831

[B34] Pang Q Christianson TA Koretsky T Carlson H David L Keeble W Faulkner GR Speckhart A Bagby GC Nucleophosmin Interacts with and Inhibits the Catalytic Function of Eukaryotic Initiation Factor 2 Kinase PKR J Biol Chem 2003 278 41709 41717 10.1074/jbc.M301392200 12882984

[B35] Chang HW Watson JC Jacobs BL The E3L gene of vaccinia virus encodes an inhibitor of the interferon-induced, double-stranded RNA-dependent protein kinase Proc Natl Acad Sci USA 1992 89 4825 4829 10.1073/pnas.89.11.4825 1350676 PMC49180

[B36] Reis SA Damaso CRA Moussatche N Vaccinia virus (strain IOC) putative double-stranded RNA binding protein Submitted (MAY-2005) to the EMBL/GenBank/DDBJ databases 2005 Cited for: NUCLEOTIDE SEQUENCE

[B37] Merkwirth C Dargazanli S Tatsuta T Geimer S Löwer B Wunderlich FT von Kleist-Retzow J-C Waisman A Westermann B Langer T Prohibitins control cell proliferation and apoptosis by regulating OPA1-dependent cristae morphogenesis in mitochondria Genes Dev 2008 22 4 476 488 10.1101/gad.460708 18281461 PMC2238669

[B38] Fusaro G Dasgupta P Rastogi S Joshi B Chellappan S Prohibitin induces the transcriptional activity of p53 and is exported from the nucleus upon apoptotic signaling J Biol Chem 2003 278 48 47853 47861 10.1074/jbc.M305171200 14500729

[B39] Lutz-Nicoladoni C Letschka T Leitges M Villunger A Baier G Essential role of PKC[delta] in apoptosis induction of mouse thymocytes Am J Immunol 2005 1 1 14 20 10.3844/ajisp.2005.14.20

[B40] Curs S Rufini A Stagni V Condò I Matafora V Bachi A Bonifazi AP Coppola L Superti-Furga G Testi R Barilà D *Src kinase *phosphorylates Caspase-8 on Tyr380: a novel mechanism of apoptosis suppression EMBO J 2006 25 1895 1905 10.1038/sj.emboj.7601085 16619028 PMC1456929

[B41] The MGC Project Team The status, quality, and expansion of the NIH full-length cDNA project: the Mammalian Gene Collection (MGC) Genome Res 2004 14 2121 2127 15489334 10.1101/gr.2596504 PMC528928

[B42] Emoto K Toyama-Sorimachi N Karasuyama H Inoue K Umeda M Exposure of Phosphatidylethanolamine on the Surface of Apoptotic Cells Exp Cell Res 1997 232 2 430 434 10.1006/excr.1997.3521 9168822

[B43] Odabaei G Chatterjee D Jazirehi AR Goodglick L Yeung K Bonavida B Raf-1 kinase inhibitor protein: structure, function, regulation of cell signaling, and pivotal role in apoptosis Adv Cancer Res 2004 91 169 200 15327891 10.1016/S0065-230X(04)91005-6

[B44] UniProtKB/Swiss-Prot P20535 (VFUS_VACCC) Integrated into UniProtKB/Swiss-Prot 1991

[B45] Goebel SJ Johnson GP Perkus ME Davis SW Winslow JP Paoletti E The complete DNA sequence of vaccinia virus Virology 1990 179 247 266 10.1016/0042-6822(90)90294-2 2219722

[B46] Golaz O Hughes GJ Frutiger S Paquet N Bairoch A Pasquali C Sanchez J-C Tissot J-D Appel RD Walzer C Balant L Hochstrasser DF Plasma and red blood cell protein maps: update 1993 Electrophoresis 1993 14 1223 1231 10.1002/elps.11501401183 8313871

[B47] Manes NP Estep RD Mottaz HM Moore RJ Clauss TR Monroe ME Du X Adkins JN Wong SW Smith RD Comparative proteomics of human monkeypox and vaccinia intracellular mature and extracellular enveloped virions J Proteome Res 2008 7 3 960 968 10.1021/pr070432+ 18205298 PMC2517256

[B48] Xu Y Hortsman H Seet L Wong SH Hong W SNX3 regulates endosomal function through its PX-domain-mediated interaction with PtdIns(3)P Nat Cell Biol 2001 3 658 666 10.1038/35083051 11433298

[B49] Gieselmann R Kwiatkowski DJ Janmey PA Witke W Distinct biochemical characteristics of the two human profilin isoforms Eur J Biochem 1995 229 3 621 628 10.1111/j.1432-1033.1995.tb20506.x 7758455

[B50] Görg A Obermaier C Boguth G Harder A Scheibe B Wildgruber R Weiss W The current state of two-dimensional electrophoresis with immobilized pH gradients Electrophoresis 2000 21 1037 1051 10.1002/(SICI)1522-2683(20000401)21:6<1037::AID-ELPS1037>3.0.CO;2-V 10786879

[B51] Laemmli UK Cleavage of structural proteins during the assembly of the head of bacteriophage T4 Nature 1970 227 259 680 685 10.1038/227680a0 5432063

